# Quantitative analysis of the distribution and mixing of cellulose nanocrystals in thermoplastic composites using Raman chemical imaging†

**DOI:** 10.1039/c8ra06674d

**Published:** 2018-10-19

**Authors:** Anna E. Lewandowska, Nor H. Inai, Oana R. Ghita, Stephen J. Eichhorn

**Affiliations:** College of Engineering, Mathematics & Physical Sciences, University of Exeter North Park Road Exeter EX4 4QF UK al18542@bristol.ac.uk; Bristol Composites Institute (ACCIS), Department of Aerospace Engineering, University of Bristol Queen's Building, University Walk Bristol BS8 1TR UK s.j.eichhorn@bristol.ac.uk +44(0) 117 33 15650

## Abstract

Cellulose nanofibers hold much promise for enhancing the mechanical properties of composites owing to their uniquely high stiffness and strength. One major issue limiting this performance however is the dispersion and mixing of cellulose nanofibers within thermoplastic resins. A combination of Raman imaging and chemical analysis has been used to quantify the distribution and mixing of cellulose nanocrystals (CNCs) in a polyethylene-matrix composite. Large area spectral imaging provides information about the effect of a compatibilizer – namely poly(ethylene oxide) (PEO) and maleated polyethylene (MAPE) – on the distribution of CNCs in the thermoplastic matrix. High-resolution images enable quantification of the degree of mixing between the CNCs and HDPE. Lower resolution images, but with greater spatial spread, allow quantification of the distribution of the CNCs. It is shown that the CNCs tend to agglomerate, with little increase in distribution even with the use of the compatibilizer. A shift in the position of characteristic Raman bands indicates the formation of hydrogen bonding between the PEO compatibilizer and the CNCs, which in turn is thought to affect the distribution of aggregates of the reinforcing phase.

## Abstract 2

Raman chemical imaging is presented to both quantify the dispersion and the degree of mixing in a cellulose nanocrystal composite.

## Graphical abstract

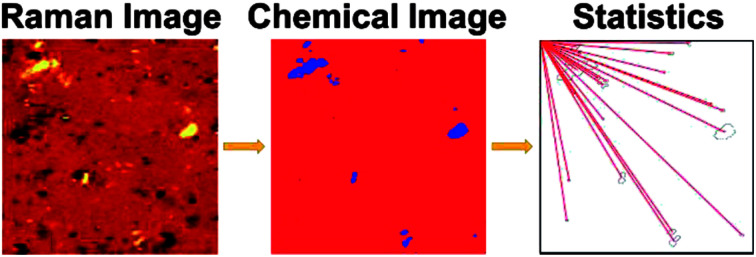

## Introduction

A growth in the economic awareness and concerns over environmental issues has triggered an increasing interest in renewable and sustainable materials. Within this grouping of sustainable materials, natural fiber composites have undergone a renaissance. In the last few decades, significant progress has been made on the production of thermoplastic polymer composites based on renewable materials and thermoplastic biodegradable polymers.^1–4^ Cellulose-based nanofibers, such as cellulose nanocrystals (CNCs) have been highlighted as promising cost effective and renewable fillers for these composite materials.^1,3,5–10^ This type of filler exhibits low density, biodegradability, high specific strength and modulus. They are also relatively easy to process and have reactive surfaces available for grafting chemical groups.^1^ Physical property advantages of cellulose-based nanofibers include their high aspect ratio (length/thickness ratio) and surface to volume ratios. These properties enable the reinforcement of composite matrices through efficient stress-transfer. It has however been generally observed that aggregation inhibits this stress-transfer process.

The hydrophilic character of cellulose-based fillers is a major disadvantage for their combination with hydrophobic thermoplastic matrices. In the absence of water, cellulose's hydrophilic nature increases its tendency to aggregate; this affects the distribution of cellulosic fillers in thermoplastic matrices. Additionally, this aggregation ultimately diminishes the effective aspect ratio of the filler, which directly influences interfacial adhesion, resulting in a reduced stress transfer efficiency. Attempts to enhance the compatibility between cellulose and thermoplastics have focused on surface chemical modification of the filler's surface^11–14^ or on increasing the surface polarity of the matrix.^3,8–10,14^ Functionalization of cellulose surfaces requires a chemical reaction between the functional groups of the modifying species and the hydroxyl groups of cellulose, and usually occurs in the presence of solvents.^12,14^ This need for chemical modification could cause difficulties for, and cost implications on, the industrial scale-up of any process. The use of compatibilizers, such as maleic anhydride grafted thermoplastics (including maleic anhydride grafted polyethylene, MAPE)^2,3,8–10,14^ and poly(ethylene oxide) (PEO)^5–7^ to modify the surface polarity of matrix are thought to be potentially more appropriate for industrial based technologies (extrusion, injection molding *etc.*).

Given that the properties of reinforced composites depend on the dispersion/distribution (*i.e.* lack of aggregation) of the reinforcing phase within the matrix,^1,15^ it is important that this property is better understood to improve performance. Independent of the approach taken for the preparation of cellulose-based composites, a method to quantify the distribution and mixing of the reinforcing phase is lacking. The efficacy of the mixing and dispersion of cellulose-based fillers into thermoplastics has typically been evaluated based on the composites' mechanical and physicochemical properties (*e.g.* crystallinity).^1–3,5–16^ The mechanical properties of composites have been widely reported using standard testing^2,3,5,7–9,12,13^ and dynamic mechanical analysis.^9,11–13^ Generally, the quality of cellulose–thermoplastic interfaces has been indirectly deduced from a comparison of the performance between neat polymer matrices and cellulose filler-containing composites. The morphologies of cellulose–thermoplastic composites have also been evaluated using microscopy *e.g.* scanning electron microscopy (SEM).^1,3,6–8,10,11^ These approaches permit the analysis of a large area of the specimen. Limitations on the size of fillers that can be resolved (submicron scale), and the resolution between components in a composite make it often difficult to quantify useful parameters such as the degree of dispersion and mixing. Other techniques, such as atomic force microscopy (AFM) have been found to be useful for morphological studies of cellulose–thermoplastic composites at the nanometer scale.^1,7,11,13^ Although AFM images can give nanoscale resolution, the evaluated area of the material is small in comparison with the size of typical specimens required for mechanical tests. Therefore, the correlation between these types of results is not always reliable. Neither of these techniques enables a chemically sensitive quantitative estimation of the distribution of cellulose fillers nor the degree of mixing between cellulose, compatibilizer and thermoplastic matrices.

Substantial developments in technology has stimulated progress in Raman imaging since the late 1990s. One such development, chemical imaging, combines structural and chemical “fingerprints” with the digital visualization of optical microscopy. This permits the identification of inorganic and organic substances, so that their quantitative or semi-quantitative amounts can be determined, as well as their size and shape in two or three spatial dimensions.^17,18^ The use of confocal microscopy restricts out-of-focus Raman scattering, reduces the fluorescent background and improves the resolution of the images obtained. Raman imaging can be performed directly on dry/wet samples or *in situ* (under different temperature/pressure conditions) without any additional sample preparation.^17,18^ The spatial resolution of the images obtained are limited by the size of the laser spot, which depends on the laser wavelength, objective lens and associated optics.^17,18^ Raman imaging has been used to determine biomaterial degradation *in vivo*,^19^ in the pharmaceutical industry to estimate the distribution of drug components in hot-melt co-extrudates,^20^ in engineering to characterize multilayer films,^21^ and in biology to study the composition of plant cell walls.^22,23^ The use of Raman imaging for the characterization of thermoplastics has focused on studies of polymer blends, since they are often not thermodynamically miscible.^24–26^ In early work, Schaeberle *et al.* used the technique to study polypropylene/polyurethane blends to understand their morphology.^24^ Later, Raman imaging was used to evaluate the quality of immiscible polymer blends.^25,26^ The spatial distribution, or the degree of mixing in the polymer blends, was evaluated by making an estimation of the domain size of the immiscible component of the blends and by a visual assessment of the domains' distribution from chemical images. None of these studies provided a statistical evaluation of the blends. In recent studies, there has been an increase in the use of imaging methodologies to study composite materials containing cellulose as a filler.^27–29^ These studies have focused on the distribution of CNCs in a polypropylene matrix composite,^27^ the intermolecular interaction between acetylated nanocrystalline cellulose (AC-NNC) and polylactic acid (PLA)^28^ and the dispersion efficiency of additives.^29^

In the present study, Raman imaging combined with chemical images are used to quantify the distribution of CNCs in high-density polyethylene (HDPE) composites. Composites were prepared using maleic anhydride modified polyethylene (MAPE) and poly(ethylene oxide) (PEO) as a compatibilizer by a melt-compounding process. Image analysis of compounded samples provides a quantitative evaluation of the distribution of the filler (low resolution imaging) and the degree of mixing (high resolution imaging) between the components. In addition to this, a chemical analysis of the Raman spectra gives evidence for a hydrogen bonding interaction between the CNCs and the PEO matrix.

In preliminary work we showed that the degree of mixing between a thermoplastic and cellulose nanocrystals (CNCs) could be quantified using this approach.^30^ In the present paper we extend this approach and show that the technique is useful for monitoring both the effect of a compatibilizer and for quantifying the distribution of fillers in a thermoplastic matrix. Such an extensive quantification of thermoplastic–cellulose nanofiber composition using Raman spectroscopy has not been previously published and could form the basis of in process monitoring of nanomaterials distribution and degree of mixing.

## Experimental section

### Materials and composites preparation

Freeze-dried CNCs were purchased from the University of Maine, Process Development Centre; USA. Sulfuric acid (purity 98%) and poly(ethylene oxide) (PEO) with a molecular weight of 5 × 10^6^ g mol^−1^ were purchased from Sigma Aldrich Inc. High density polyethylene (Arboblend HDPE; molecular weight = 1.33 × 10^5^ and melt volume flow rate = 20) was supplied by Tecnaro GmbH, while maleated polyethylene (A-C 575A, MAPE copolymer) was provided by Honeywell.

Two sets of samples were compounded with CNCs loadings of 0.62, 1.25, 2.50 and 5.00 wt% for CNCs/MAPE/HDPE and 0.50, 1.50, 2.50 and 5.00 wt% for CNCs/PEO/HDPE. The composites were prepared by a compounding and extrusion process following the procedures described previously by Lewandowska and Eichhorn^30^ for composites containing MAPE as a compatibilizer, and by Inai *et al.*^5^ for composites containing PEO. Freeze-dried CNCs were used, as purchased, for the preparation of CNCs/MAPE/HDPE samples, while the CNCs-PEO material was prepared from aqueous solutions using a freeze-drying process prior to the compounding of CNCs/PEO/HDPE samples. The filler, compatibilizer and matrix were mixed in a mortar for 8 min. The mixture was then dried in a vacuum oven at a temperature of 60 °C for 24 h to remove humidity. The composites were melt-compounded in a counter rotating twin-screw extruder (HAAKE Rheomex CTW5, Thermo Fisher Scientific) at a temperature of 160 °C. The composites were then extruded as filaments (*ø* ∼ 2 mm) after mixing for 7 min at a speed of 70 rpm. Prior to imaging, the composite filaments were cryo-microtomed into slices with a ∼20 μm thickness.^30^

### Large area confocal Raman imaging for filler distribution

A preliminary development of Raman imaging methodology for quantitative analysis of composite samples has been previously described by Lewandowska and Eichhorn.^30^ Large area Raman imaging (LARI) was performed using a confocal Raman microscope. An Alpha300 (WITec GmbH), equipped with a UHTS 300 VIS-NIR spectrometer optimized for NIR excitation and a thermoelectrically cooled CCD detector (down to −61 °C) was used. This system contained a 600 g mm^−1^ grating blazed (BLZ) at 750 nm. A 785 nm wavelength laser (NIR) was used for excitation of the Raman scattering, and a 50× objective lens was used for the backscattered light collection with a lateral resolution of 684 nm. The laser power at the sample was 41 mW. Raman imaging measurements were performed on cryo-microtomed cross sections of the composites. Images were recorded from an area of 200 × 200 μm^2^ (40 000 μm^2^) with a step size of 2 μm in both the *x*- and *y*- directions, using an exposure time of 4 s. A total 10 000 Raman spectra were recorded for each image. At least three images per composite sample were used in the analysis.

WITec Project Plus software was used to analyze Raman images and to convert them into chemical images. These chemical images were subsequently analyzed using Image-J software to estimate distances between a fixed reference point in an arbitrary coordinate system and the center of mass related to the regions containing CNCs, and their area. Extraction of the objects using Image-J was performed using an automated threshold with the algorithm ‘IsoData’.

### High resolution confocal Raman imaging for mixing of components

High resolution Raman imaging (HRRI) was performed on the same confocal Raman microscope used for the LARI analysis, equipped with a UHTS 300 VIS spectrometer. This system featured a thermoelectrically cooled CCD detector (down to −62 °C), with a grating 600 g mm^−1^ (BLZ = 500 nm). A 532 nm wavelength laser (VIS) was used for excitation, and a 50× objective lens was used for the backscattered light collection. The lateral resolution, using this set-up was ∼388 nm. The laser power on the sample was 10 mW. HRRI was performed on the same composite cross sections as for the LARI measurements. Raman images were recorded within an area of 50 × 50 μm^2^ (2500 μm^2^) with a step size of 0.2 μm in both the *x*- and *y*- directions with an exposure time of 0.2 s. A total of 62 500 Raman spectra were recorded for each image. A minimum of four images per composite were used for image analysis. Single Raman spectra from reference compounds were recorded using an exposure time of 1 s and fifty accumulations. The same image analysis procedure that was used for LARI analysis was utilized for these measurements, although only the percentage of the area related to each component in the chemical image was determined.

## Results and discussion

Quantitative analysis of the filler distribution in a volume of matrix, and the degree of mixing between these components using Raman and chemical imaging require their unambiguous differentiation. Typical reference Raman spectra for HDPE, PEO, CNCs and CNCs-PEO are shown in Fig. S-1 (ESI†). Verification of the presence of CNCs is confirmed by three distinct Raman bands located at ∼382 cm^−1^, ∼1100 cm^−1^ and ∼1381 cm^−1^. The Raman bands for CNCs significantly overlap with those from PEO (Fig. S-1a and S-1b†). The Raman band centered at ∼382 cm^−1^ falls within the region 250–600 cm^−1^, which has been assigned to skeletal-bending modes involving the CCC, COC, OCC, OCO and skeletal stretching modes of CC and CO moieties.^31^ The most intense Raman band, characteristic for the CNCs, is located at ∼1100 cm^−1^ and has been previously assigned to the CO ring and the β-1,4 glycosidic linkage (COC) stretching modes between the glucose rings of the cellulose chains.^31,32^ The Raman band located at ∼1381 cm^−1^ falls within the region 1180–1550 cm^−1^, which has been assigned to bending modes involving the CCH, OCH and COH moieties.^31^ The Raman spectrum for PEO is richer in Raman bands than HDPE (Fig. S-1c and S-1d†). Two distinctive Raman bands located at ∼847 cm^−1^ and ∼864 cm^−1^ correspond to CH_2_ rocking modes and to a COC stretching mode, respectively.^33,34^ Furthermore, Raman bands centered at ∼1285 cm^−1^ and ∼1483 cm^−1^, assigned to CH_2_ twisting modes and CH_2_ scissoring modes, were additionally used in the analysis to distinguish PEO and HDPE.^33,34^ Verification of the presence of HDPE matrix is based on the appearance of unique Raman bands located at ∼1301 cm^−1^ and ∼1464 cm^−1^, which have been assigned to CH_2_ twisting modes and CH_2_ rocking modes in the crystalline phase of the polymer.^25,35,36^

### Quantitative analysis of the filler distribution

**Fig. 1:**
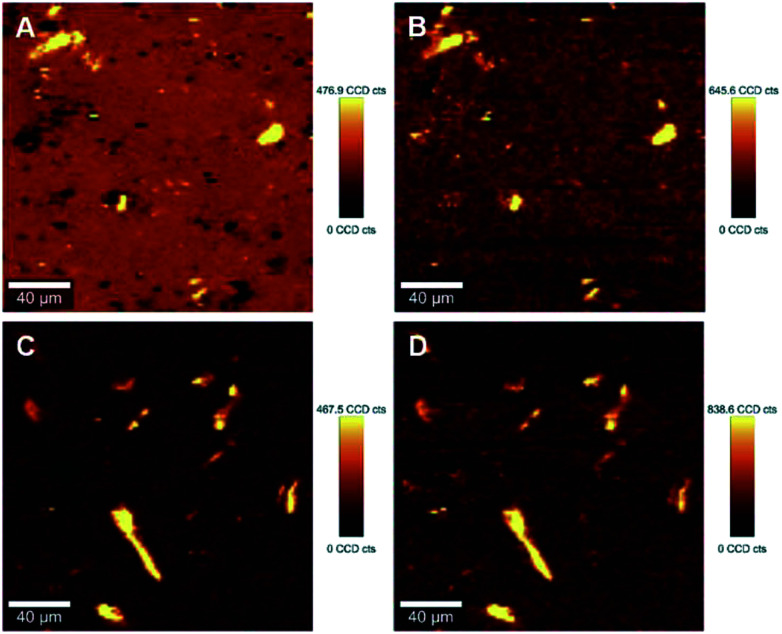
Typical large area Raman images (LARI) of 1.25% CNCs/MAPE/HDPE (A and B) and 1.50% CNCs/PEO/HDPE (C and D) composites depicting the intensity of Raman bands located at ∼382 cm^−1^ (A and C) and ∼1100 cm^−1^ (B and D). The scales to the right of the images are in counts (cts).

**Fig. 2:**
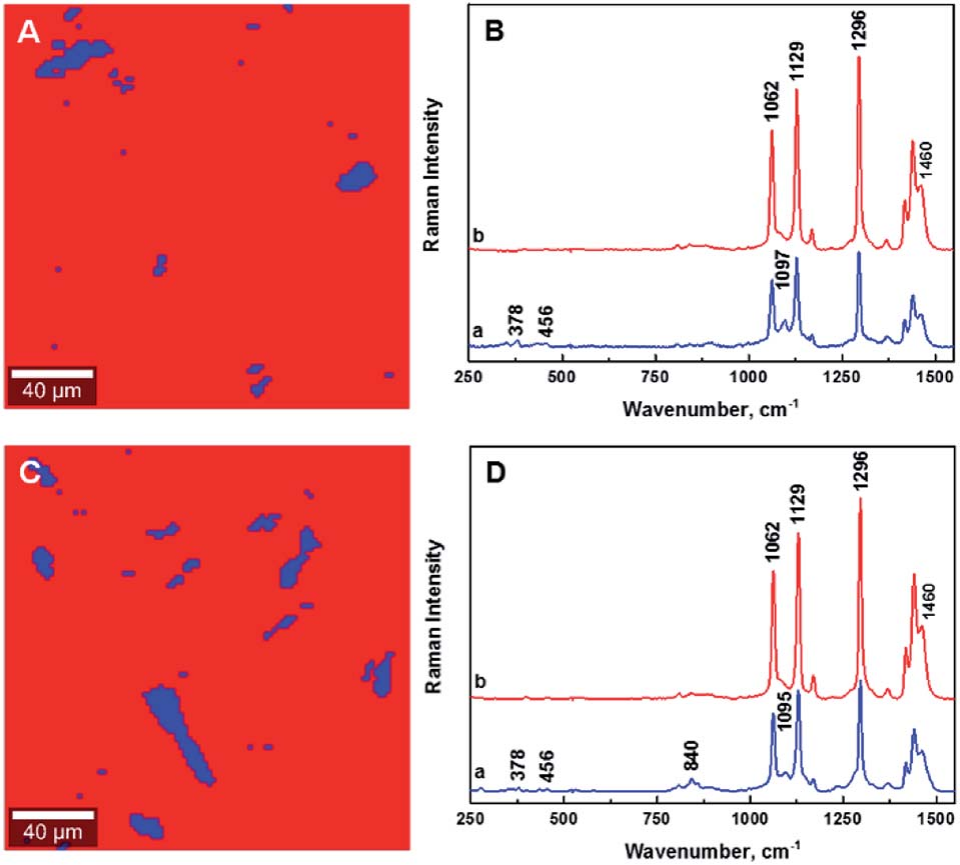
Typical chemical images for 1.25% CNCs/MAPE/HDPE (A) and 1.50% CNCs/PEO/HDPE (C) composites depicting the general chemical composition of a mapped cross-section on Fig. 1, where CNCs are depicted in blue and HDPE in red. Typical Raman spectra of composite components in the chemical images for 1.25% CNCs/MAPE/HDPE (B) and 1.50% CNCs/PEO/HDPE (D).

The ability to image the distribution of the fillers is the aim of the present study. The spatial resolution of the technique however allows the mapping of the distribution of aggregates of a certain size and does not permit the imaging of individualized filler particles; in this case CNCs. The smallest aggregates detected in the images have a size of ∼2 μm^2^, which is resolvable using the laser with the lateral resolution of the laser spot ∼684 nm and a step size of the measurement 2 μm. Nevertheless, both the distribution of CNC aggregates within the HDPE matrix, and their sizes can be estimated using LARI. Fig. 1 illustrates some typical outputs of LARI for CNCs/MAPE/HDPE and CNCs/PEO/HDPE composites; Fig. 2 shows their corresponding chemical images. The distribution of cellulose is shown by Raman images depicting the intensity of two unique Raman bands located at ∼382 cm^−1^ and ∼1100 cm^−1^ (Fig. 1). A bright yellow color relates to the highest intensity of the selected Raman bands, while a brown or a deep brown color correspond to regions where these bands show a significantly low intensity or are not detected. The yellow areas observed in Fig. 1, as well as Fig. S-2 and S-3 (ESI†), confirm the presence of CNCs in the scanned area. In all images CNCs are present as “islands” in the polyethylene matrix suggesting extensive aggregation. The shapes of these “islands” are irregular, independent of the presence of MAPE or PEO compatibilizers. The larger aggregates with an area greater than 400 μm^2^ appear more frequently at higher loadings of CNCs (see distributions in Fig. S-2, S-3, S-4A and B, ESI†).

Fig. 2 shows typical chemical images of CNCs/MAPE/HDPE and CNCs/PEO/HDPE composites derived from Fig. 1, accompanied by Raman spectra of the composite components distinguished in these images. The chemical images are the graphical representation of the chemical composition of Raman images, as described previously.^30^ The red area in the chemical images, is characteristic of the HDPE, because a corresponding Raman spectrum shows only the spectral features characteristic of polyethylene (also colored red). Areas rich in CNCs are indicated by the blue color in the chemical images with a corresponding Raman spectrum also colored in blue. Their size and distribution confirm the tendency of the CNCs to form aggregates. Nevertheless, the intensity of the most intense cellulose Raman band located at ∼1095 cm^−1^ is significantly low compared to a characteristic HDPE band (at ∼1296 cm^−1^) (Fig. 2B and D). This low intensity arises from a lower resolution of the large area Raman images, which are recorded with a step size of 2 μm. A lower resolution of imaging results in a lower detection sensitivity, and so there is a chance that some smaller cellulose aggregates will be omitted during the measurements.

**Fig. 3:**
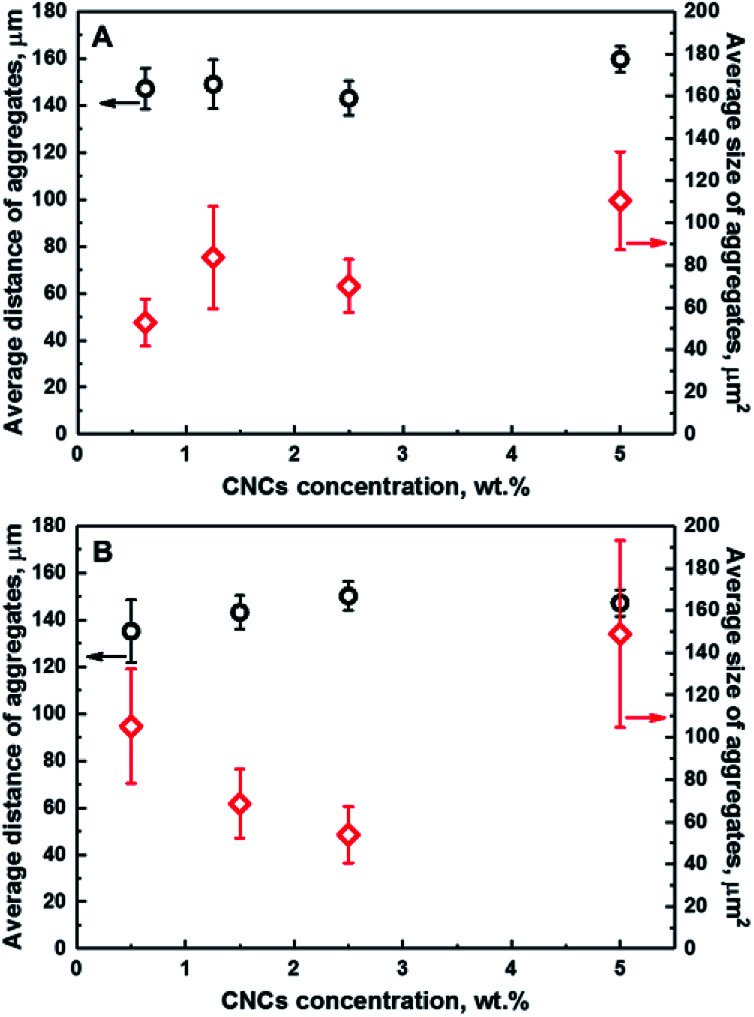
Dependence of the average distance of aggregates from a fixed point in a coordinate system (black) and the average size of aggregates (red) on the concentration of CNCs for CNCs/MAPE/HDPE (A) and CNCs/PEO/HDPE (B) composite samples.

Fig. 3 illustrates the dependence of the average distance of aggregates from a fixed point in the coordinate system to their centers of mass, and the average size of CNCs aggregates as a function of the CNCs loading for series of composites; namely CNCs/MAPE/HDPE (A) and CNCs/PEO/HDPE (B). These variables provide a quantitative description of the distribution of CNCs in the HDPE matrix. Both quantities are estimated from chemical images using ImageJ software. A schematic representation of the ImageJ analysis is illustrated in Fig. S-5 (ESI†). The exact values of those variables are given in Table S-1 (ESI†). There are large variations in the error values, especially for the average size data, indicating a considerable variability in the size of the aggregates. No correlation between the average distance and size of CNCs aggregates is observed for composites prepared using maleated polyethylene as a compatibilizer (CNCs/MAPE/HDPE) (Fig. 3A). Both of these variables are independent of the cellulose loading. In contrast to this, the samples made with the PEO compatibilizer (CNCs/PEO/HDPE) exhibits a dependence between the distribution and size of CNCs aggregates (Fig. 3B). The average distance of the aggregates from an arbitrary reference point increases from ∼135 μm for 0.50 wt% to ∼150 μm for 2.50 wt%, and subsequently stays within the same range of values for 5.00 wt% (Table S-1, ESI†). Simultaneously, the average size of aggregates decreases between cellulose loadings of 0.50 wt% and 2.50 wt%, and increases significantly from ∼54 μm^2^ for 2.50 wt% to ∼149 μm^2^ for 5.00 wt% (Table S-1, ESI†). The samples containing PEO exhibit an increase in the average distance of aggregates between CNCs loadings of 0.50 wt% and 2.50 wt%, while the average size of aggregates shows an inverse ‘volcano curve’ for the same loading (Fig. 3B). This shows that there is a correlation between the distance and the size of CNCs aggregates. Though, the spatial resolution of the Raman images is limited by the optics – the detection optics as well as the focusing optics – we cannot exclude the underlying dispersion of smaller aggregates below the detection limit of the technique. The values of the average distance of the aggregates increases with a simultaneous reduction in their average size; this is an indication of a change in the ‘evenness’ of the CNCs' distribution in this composite sample. It appears that the optimum loading of CNCs in the PEO compatibilized composites is close to 2.50 wt%; above this loading only a growth of the aggregates is observed.

At high levels of CNC loading (>5%) the error bars are significantly large, particularly for the CNCs/PEO/HDPE samples. This large error in the data suggests that there is a significant spread of aggregate sizes. A similar Raman imaging methodology has been employed for studying the dependence of the average size of graphene platelets in an epoxy matrix.^37^ A step change in the average agglomerate size was observed at a certain loading, indicating the presence of an optimum concentration for a homogeneous, smaller aggregate size distribution.^37^ The influence of the CNCs loading on the mechanical properties have been previously reported for unmodified and PEO-modified CNCs/HDPE composites.^5^ Initially, the tensile modulus and strength of composites increased with an increasing CNCs content up to 1.5 wt%. Above this value a continuous decrease in tensile modulus and strength was reported. Additionally, an improvement in the mechanical properties was demonstrated for PEO-modified CNCs/HDPE composites.^5^

**Fig. 4:**
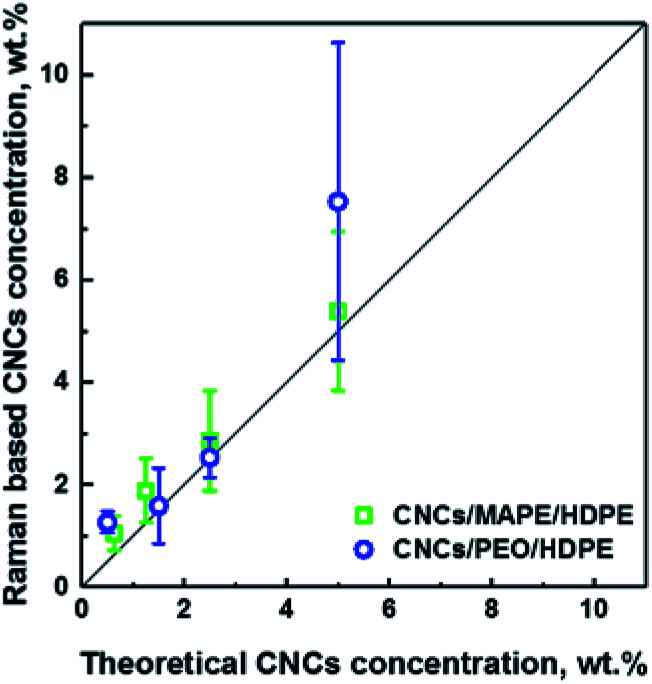
Dependence of the Raman based CNCs concentration on the theoretical concentration of CNCs used in the preparation of CNCs/MAPE/HDPE and CNCs/PEO/HDPE composite samples. The solid straight line represents a 1 : 1 relationship; an *R*^2^ value of 0.95 was obtained between these data and the line.

Additionally, LARI images have been used to estimate the concentration of CNCs in the samples (see Fig. 4). The concentration of the CNCs was been estimated from the chemical images using ImageJ software, which allows a calculation of the total area of the aggregates in the image. This area of the aggregates was then divided by the CNCs' density (a value of 1.5 g cm^−3^ was used). The values of Raman based CNC concentrations correlate well (*R*^2^ = 0.95) with the 1 : 1 line (solid black line) which suggests that there is an aggregation of CNCs in all samples independent of the original loading. This correlation also provides confidence that the Raman images give an accurate representation of the CNC fraction in the composites. At 5 wt% there is a large error bar associated with a the PEO/CNCs. This increase in the scatter of the data is thought to be due to agglomeration and an increase in width of the distribution of aggregate sizes.

### Degree of mixing of composites

**Fig. 5:**
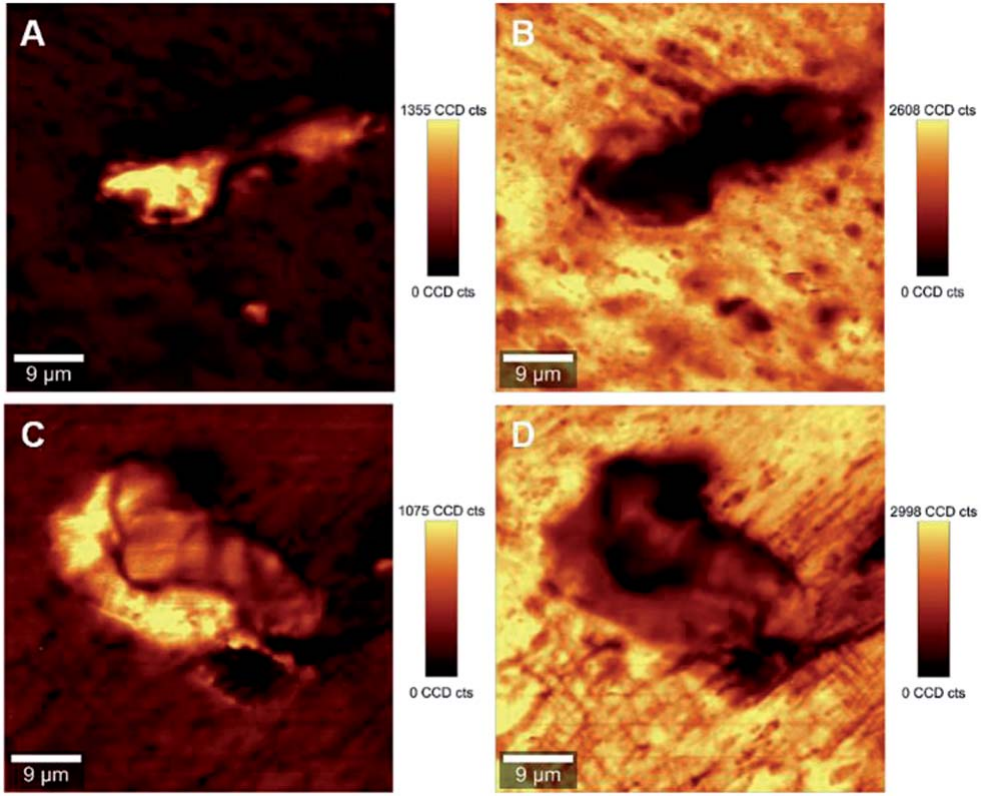
Typical high-resolution Raman images (HRRI) of 1.25% CNCs/MAPE/HDPE (A and B) and 1.50% CNCs/PEO/HDPE (C and D) composites depicting the intensity of Raman bands located at ∼1100 cm^−1^ (A and C) and ∼1301 cm^−1^ (B and D).

Interfacial adhesion is another critical factor influencing the properties of reinforced composites; this adhesion typically results in an increased stress transfer efficiency. Interfacial adhesion is likely to be affected by the degree of mixing between the matrix and the reinforcing phase; where a low level of mixing takes place, little adhesion is also likely to occur. The quantitative evaluation of a ‘degree of mixing’ of CNCs-based composites can be assessed using HRRI. Fig. 5 (also Fig. S-6 and S-7, ESI†) demonstrates typical outputs from HRRI of CNCs/MAPE/HDPE and CNCs/PEO/HDPE composite samples. These images demonstrate the high chemical sensitivity used for the assessment of the role of the compatibilizer and its interaction with the CNCs. Despite this high-resolution capability, it is not possible to discriminate single CNCs on the nanometer scale (typically CNCs are 98 ± 21 nm long and 2.3 ± 0.17 nm in diameter; data based on TEM images – not shown) because of the limitations of the lateral resolution of the laser spot (∼388 nm). These images present additional features of the surface morphology associated with the presence of cellulose aggregates and the process of microtome cutting. The same color scale was used for the high-resolution images, as for the LARI; a bright yellow color corresponds to the highest intensity of the selected Raman bands, while a deep brown color corresponds to areas where these bands show a significantly low intensity or are below the detection level. The bright yellow color in Fig. 5A and C indicate a high intensity attributed to the Raman band located at ∼1100 cm^−1^ (from the CNCs). The WITec Raman system uses two different optical set-ups for the LARI and HRRI. This results in two different band positions for the peak located at ∼1095 cm^−1^ (785 nm laser) and ∼1100 cm^−1^ (532 nm).

These images further reveal the tendency of CNCs to aggregate, independent of the compatibilizer used for their preparation. The cellulose aggregates form “islands” in the volume of HDPE matrix with highly random shapes. The intensity of the yellow color changes across the “islands” suggesting a variability in the concentrations of CNCs. A deep brown color surrounding the CNCs' “islands” indicates the absence of the Raman band located at ∼1100 cm^−1^, and a lack of cellulosic material in this area (Fig. 5A and C). These regions turn to a bright yellow color, when the intensity of a Raman band located at ∼1301 cm^−1^, corresponding to CH_2_ twisting mode in polyethylene, is observed (Fig. 5B and D). The intensity of the Raman band indicative of HDPE reduces to a ‘light brown’ color in the areas assigned to aggregates of CNCs. This color change indicates the existence of a region where mixing of the filler (CNCs) and matrix (HDPE) takes place.

**Fig. 6:**
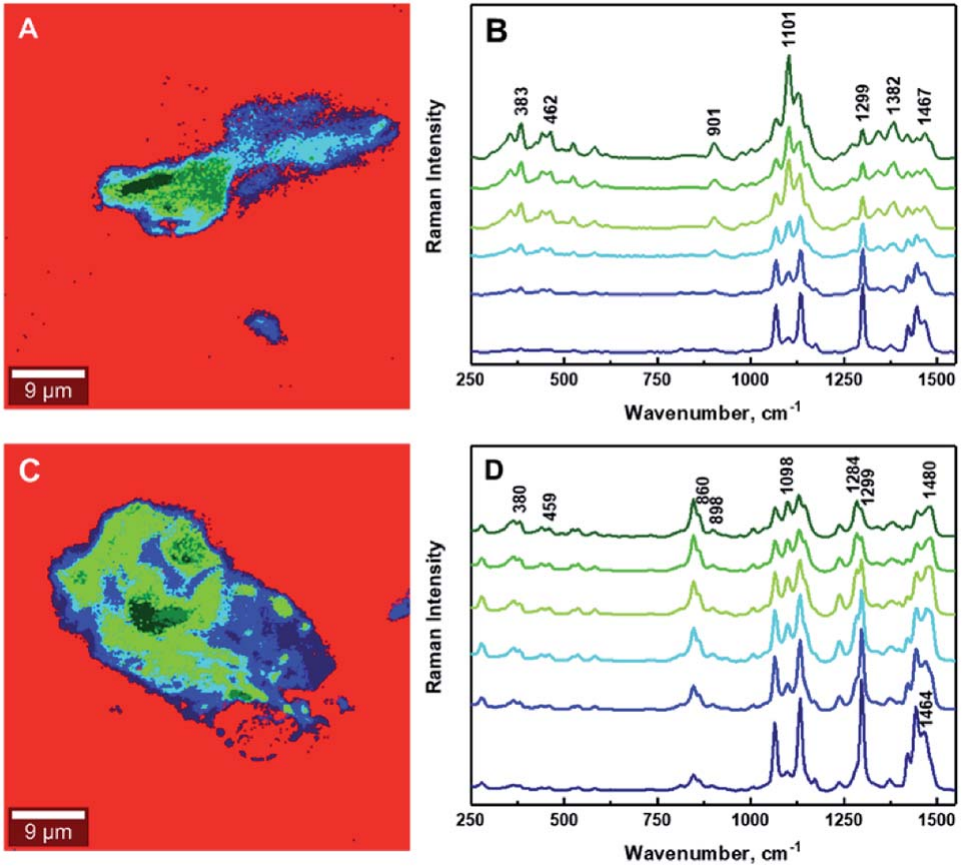
Typical chemical images of 1.25% CNCs/MAPE/HDPE (A) and 1.50% CNCs/PEO/HDPE (C) composites depicting detailed chemical compositions of a mapped cross-section taken from Fig. 5. Typical Raman spectra corresponding to mixing components within the chemical images, where (B) 1.25% CNCs/MAPE/HDPE and (D) 1.50% CNCs/PEO/HDPE.

The estimation of the boundary between the phases in the composites is critical for the quantification of the degree of mixing between the CNCs and the matrix. Fig. 6 illustrates the gradual changes in the concentration between filler, compatibilizer and matrix in the composites for the 1.25% CNCs/MAPE/HDPE and 1.50% CNCs/PEO/HDPE samples. These chemical images show spatially, the gradual changes in concentration of composite components (Fig. 6A and C), while the Raman spectra reflect the changes in the relative intensity of their respective Raman bands (Fig. 6B and D). It is important to note that the core of the aggregates contains only cellulose for the CNCs/MAPE/HDPE composites and within the CNCs-PEO phase for the CNCs/PEO/HDPE composites. Raman bands corresponding only to the presence of cellulose dominate the core of the aggregate structures in the CNCs/MAPE/HDPE composites; they overlap with the Raman bands characteristic for HDPE, apart from the most intense band for the CH_2_ twisting mode located at ∼1299 cm^−1^ (Fig. 6A and B; dark green). Additionally, in the aggregate core there are well-resolved regions of cellulose skeletal-bending modes, with intense Raman bands located at ∼383 cm^−1^ and bending modes with a band located at ∼1382 cm^−1^. The intensity of these Raman bands gradually decreases outwards from the core of the aggregates. The regions rich in cellulose are depicted with a scale of green colors; this scale changes from a ‘dark green’ to ‘lime’ color, corresponding to a reduction in the concentration of cellulose. A region of mixing between the CNCs and HPDE is noted, away from the aggregates' core, and is depicted as a blue scale of colors; this scale changes from a ‘cyan’ to a ‘navy’ color, moving out from the core of the aggregates, matching the gradual increase in the concentration of polyethylene. The existence of the most intense cellulose Raman band, located at ∼1101 cm^−1^, confirms the presence of CNCs in the mixed region (Fig. 6A and B, navy). The core of the aggregate structures for the CNCs/PEO/HDPE composite specimens consists mainly of the CNCs-PEO phase, which is confirmed by the presence of typical cellulose Raman bands located at ∼380 cm^−1^ and 1098 cm^−1^ as well as bands corresponding to CH_2_ rocking modes and to COC stretching modes located at ∼847 cm^−1^ and ∼864 cm^−1^ and the CH_2_ twisting and scissoring modes at ∼1285 cm^−1^ and ∼1483 cm^−1^ characteristic of PEO (Fig. 6C and D; dark green). The Raman band located at ∼1299 cm^−1^, which is typical of HDPE, appears as a shoulder for the Raman band of poly(ethylene oxide) (at ∼1285 cm^−1^). Changes in the concentration between the CNCs-PEO phase and the HDPE matrix were assessed using the ratio of the Raman bands typical for PEO and HDPE; namely *I*_1299/1284_ and *I*_1464/1480_. It is found that these intensity ratios gradually decrease moving out from the core to the outer edge of the aggregates. The green colors correspond to regions rich in the CNCs-PEO phase; a decrease in the concentration of this phase is reflected by a change from a deeper to a lighter color. The mixing is shown as a scale of blue colors, where the increase of the intensity of the blue color corresponds to an increase in the concentration of the HDPE matrix. The evaluation of chemical images reveals that the regions richest in CNCs or CNCs-PEO phases are relatively small compared to the aggregate size. The aggregates are dominated by regions where the transition between an area richer in CNCs (Fig. 6A and C, lime) to those abundant in HDPE (Fig. 6A and C, cyan).

It is critical to establish the boundary between aggregated and mixed phases using the ratio of Raman band intensities. The determination of the boundaries between different phases within the composites has been enabled by calculating the intensity ratio of the Raman band corresponding to polyethylene (∼1301 cm^−1^) to that of the CNCs (∼1381 cm^−1^) for CNCs/MAPE/HDPE composites, and the Raman band characteristic of poly(ethylene oxide) (∼1285 cm^−1^) for CNCs/PEO/HDPE composites (Table S-2, ESI†). The intensities of these Raman bands have been assessed by deconvolution using a Lorentzian function. The boundary between the aggregated and mixed phases in the CNCs/MAPE/HDPE and CNCs/PEO/HDPE composites are estimated to be 1.2 and 1.1 respectively, using the *I*_1301/1381_ and *I*_1301/1285_ ratios. Therefore, all regions with an intensity ratio < 1 are considered to be in an aggregated state, while those with an intensity ratio > 1 are considered to be mixed.

**Fig. 7:**
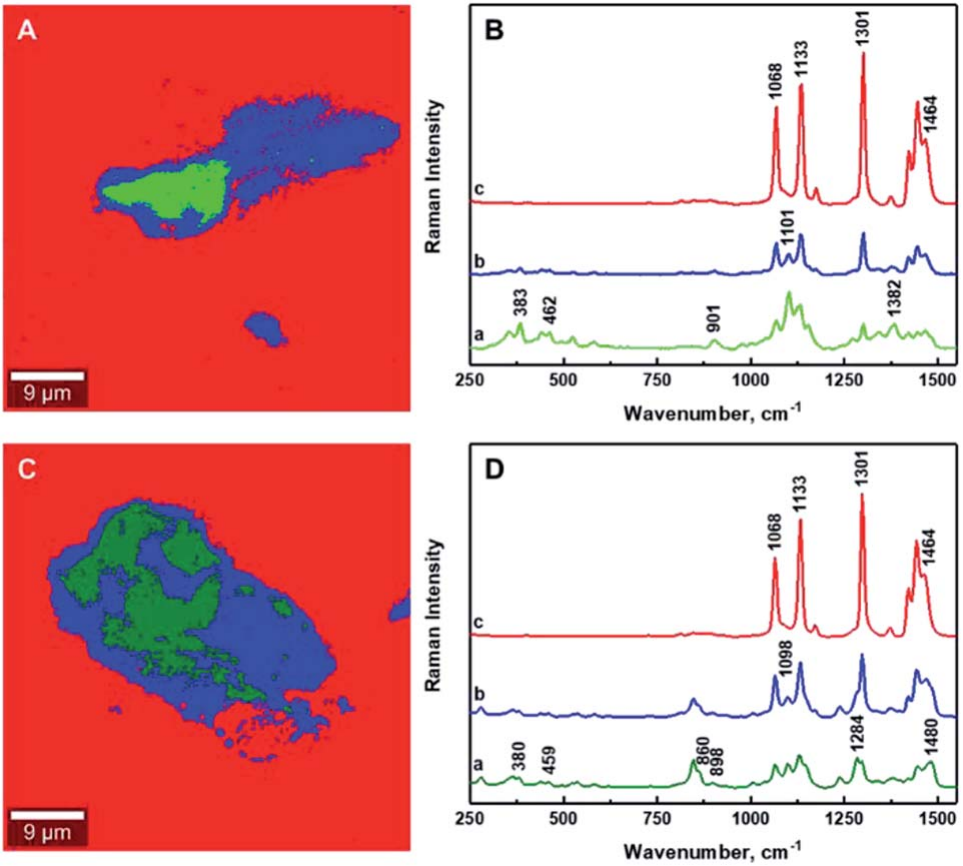
Typical chemical images of 1.25% CNCs/MAPE/HDPE (A) and 1.50% CNCs/PEO/HDPE (C) composites depicting the general chemical composition of a mapped cross-section from Fig. 5. Typical Raman spectra of bands observed within the mixing zone of the chemical images of 1.25% CNCs/MAPE/HDPE (B) and 1.50% CNCs/PEO/HDPE (D).

Fig. 7 as well as Fig. S-6 and S-7† show the chemical images extracted from the Raman images for the CNCs/MAPE/HDPE and CNCs/PEO/HDPE composites. The red color dominating these images is related to the presence of polyethylene matrix. The areas corresponding to the cellulose aggregates are depicted in green and blue colors. The boundary between the aggregated (green) and mixed (blue) regions is distinguished by intensity ratios; the *I*_1301/1381_ ratio for CNCs/MAPE/HDPE and the *I*_1301/1285_ ratio for CNCs/PEO/HDPE. The Raman spectra assigned to all three regions of the chemical images are presented in Fig. 7B and D. Their detailed analysis provides additional information related to the interfacial adhesion between the filler and compatibilizer. It is possible to detect a shift in the position of the Raman bands, which is thought to derive from an interaction between the filler and the compatibilizer. In the case of maleated polyethylene, compatibilization is expected to take place through an esterification reaction and/or hydrogen bonding between the maleic anhydride groups (–COOH and –C=O) and the hydroxyl groups (–OH) of the cellulose.^10,38^ It is assumed, that the ratio of ester linkages to hydrogen bonds depends on the ratio of the content of cyclic anhydride to dicarboxylic acid in the MAPE.^38^ The Raman spectrum of maleated polyethylene (MAPE) used in the preparation of these composite specimens only exhibits bands typical for polyethylene.^30^ Additionally, the position of Raman bands corresponding to CNCs in the CNCs/MAPE/HDPE composite is the same as for the reference spectrum for CNCs (Fig. S-1† and 7B). This suggests a minimal interfacial interaction between CNCs and MAPE. In the case of poly(ethylene oxide), an interaction is expected to occur with the CNCs *via* hydrogen bonds between the primary hydroxyls at the C6 position in cellulose, and the ether oxygen in the PEO chain.^39^ Raman bands corresponding to CNCs and PEO are found to shift towards a lower wavenumber position in the CNCs/PEO/HDPE composites (Fig. S-1† and 7D). The Raman band located at ∼864 cm^−1^, characteristic of PEO, shifts in position to ∼860 cm^−1^, while the band located at ∼847 cm^−1^ remained unchanged for the CNCs/PEO/HDPE composites. The Raman band located at ∼864 cm^−1^ corresponds to a combination of symmetric CH_2_ rocking modes and symmetric COC stretching modes.^33,34^ This vibration is therefore sensitive to hydrogen bonding interactions, since it involves the oxygen atom in the PEO backbone chain. A similar change was observed for the dissolution of poly(ethylene oxide) in water.^33^ Additionally, the formation of hydrogen bonds is also supported by a shift of the Raman band located at ∼1069 cm^−1^, which is also related to the symmetric COC stretching mode of the backbone chain.^33^ This Raman band however overlaps with the bands characteristic of HDPE and CNCs, which makes it difficult to assign its exact position. A shift in the position of the Raman bands corresponding to CNCs in the CNCs/PEO/HDPE composites is also observed (Fig. S-1† and 7D). Bands centered at ∼901 cm^−1^ and ∼1101 cm^−1^ shift towards a lower wavenumber position (by ∼3 cm^−1^). Although, these bands correspond to the vibrational modes not directly involved in the formation of hydrogen bonds, their position is sensitive to the deformation of the cellulose chains, which may arise from their formation. Additionally, there is a shift in the Raman band located at ∼382 cm^−1^, which arises from bonds that are in the same orientation as the moieties giving rise to the band located at ∼1101 cm^−1^.^40^ This shift suggests the presence of an interaction between the CNCs and PEO.

Tables S-3 and S-4 (ESI†) exhibit the areas corresponding to the matrix, the mixing of the matrix with the filler and the filler aggregation within the chemical images, as estimated using ImageJ software. The total area of the Raman images is 2500 μm^2^. The Raman images were used to quantify the degree of mixing between CNCs and HDPE using an average areal ratio of blue/(red + blue + green) and the degree of aggregation of CNCs as green/(red + blue + green). Both of those variables are independent of the cellulose loading for the CNCs/MAPE/HDPE and CNCs/PEO/HDPE samples. Additionally, the average values of both the degree of mixing and the degree of aggregation show a high standard deviation suggesting that the studied samples are highly random without statistically significant differences.

## Conclusions

The present study has demonstrated that Raman imaging is a versatile technique to investigate cellulose-based nanocomposite materials. The possibility of using large area imaging to map the distribution of CNCs, and high-resolution imaging their mixing with a thermoplastic resin has been demonstrated. Quantification of these images enables the average distance of the aggregates from a fixed point to be obtained. This distance is found to be invariant of the filler loading. The size of the aggregates is however sensitive to the filler loading. It was found to increase with filler loading for the maleated polyethylene (MAPE) samples, but initially decrease up to a 2.5 wt% CNC loading for the PEO modified CNCs. This latter result suggests that there is a small fraction of dispersed CNCs in the sample, which might be the reason why PEO is such an effective agent for increasing the mechanical properties of CNC/PE composites. There was good agreement between the CNC concentration derived from the Raman imaging measurements and the that expected from the amount of CNCs added during compounding. This suggests that the Raman imaging is a reliable and representative analysis of the composition of the samples. Raman imaging has also demonstrated that PEO has an interaction with the CNCs through hydrogen bonding, while MAPE does not appear to increase the interfacial interaction. These interactions have an impact on the dispersion of the CNCs in the HDPE matrix, leading to more uniform composites prepared with PEO as a compatibilizer. Additionally, the combination of Raman images and chemical “finger prints” from the images provide information on the mixing of HDPE with CNCs, and the agglomeration of the latter in a compounded composite. Nevertheless, both composite samples studied showed no correlation between the degree of mixing and the filler loading.

## Conflicts of interest

There are no conflicts to declare.

## Supplementary Material

RA-008-C8RA06674D-s001
