# Supplementary material for: Quantitative analysis of the distribution and mixing of cellulose nanocrystals in thermoplastic composites using Raman chemical imaging
Source: RSC Adv. 2018 Oct 19;8(62):35831–9. doi: 10.1039/c8ra06674d (PMC9088214; doi:10.1039/c8ra06674d)
Supplement: RA-008-C8RA06674D-s001 [file RA-008-C8RA06674D-s001.pdf]

Electronic Supplementary Material (ESI) for RSC Advances.
This journal is © The Royal Society of Chemistry 2018

Supporting Information for:

Quantitative Analysis of the Distribution and Mixing of Cellulose Nanocrystals in Thermoplastic Composites Using Raman Imaging

Anna E. Lewandowska[§,‡], Nor H. Inai[§], Oana R. Ghita[§] and Stephen J. Eichhorn*[,‡]

[§] College of Engineering, Mathematics & Physical Sciences, University of Exeter, North Park Road, Exeter, EX4 4QF, United Kingdom

[‡] Bristol Composites Institute (ACCIS), Department of Aerospace Engineering, University of Bristol, Queen's Building, University Walk, Bristol BS8 1TR, United Kingdom

* Corresponding Author E-mail: s.j.eichhorn@bristol.ac.uk

This file contains the supporting Figures S-1 to S-7 and supporting Tables S-1 to S-4

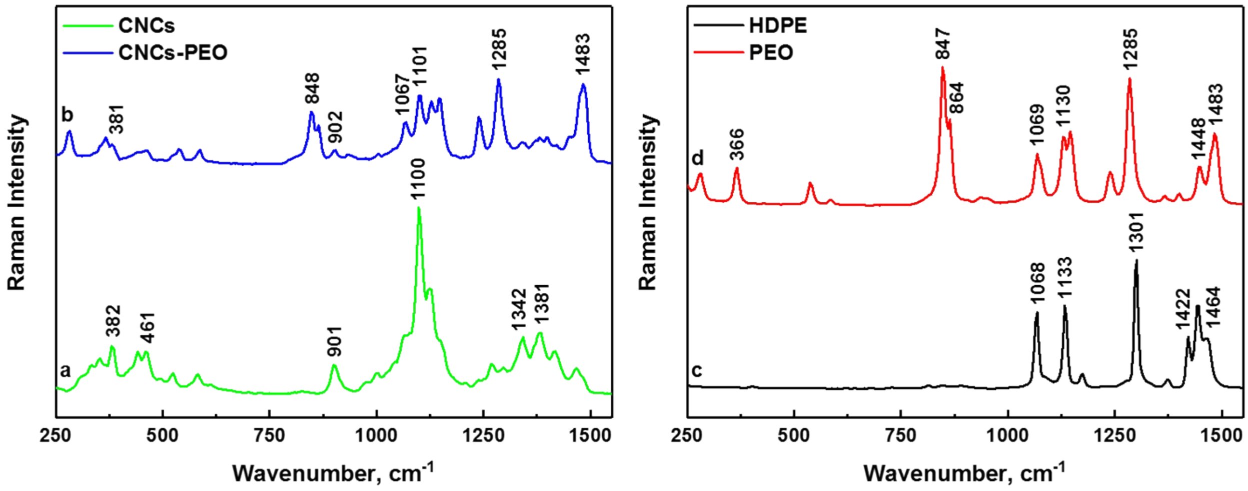

Figure S-1. Typical reference Raman spectra for: (a) freeze-dried cellulose nanocrystals (CNCs), (b) freeze-dried CNCs-PEO, (c) high-density polyethylene (HDPE) and (d) poly(ethylene oxide) (PEO).

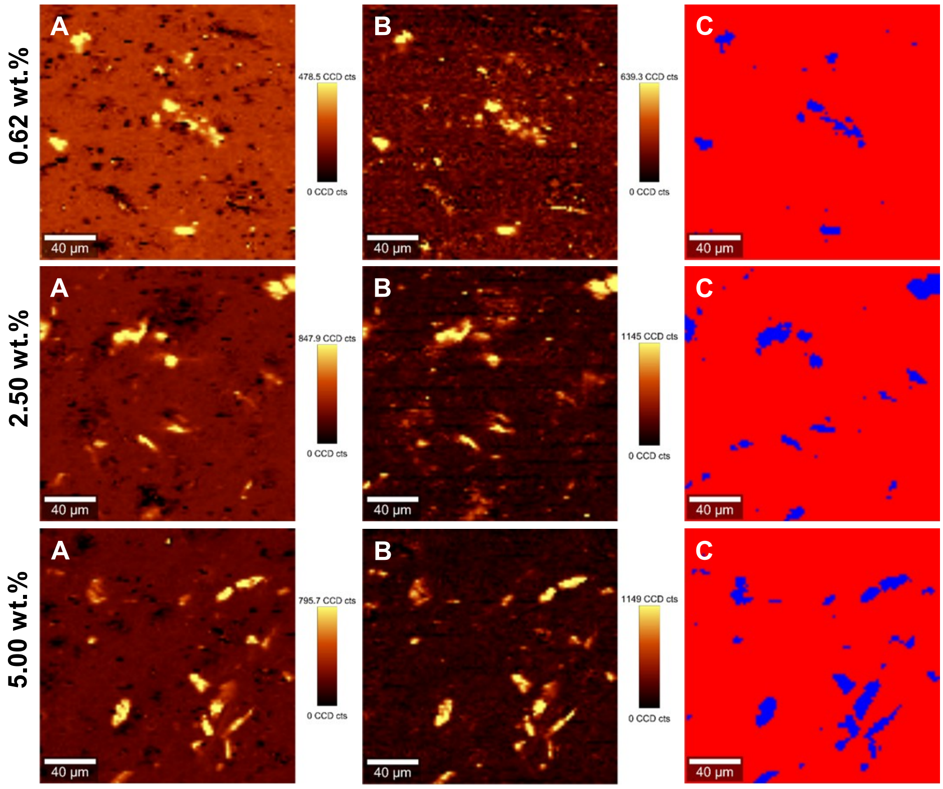

Figure S-2. Typical large area Raman images (LARI) of 0.62% (top), 2.50% (middle) and 5.00% (bottom) of CNCs/MAPE/HDPE composites depicting the intensity of Raman bands located at ~380 cm^{-1} (A) and ~1100 cm^{-1} (B). Chemical images of studied area (C) showing HDPE in red and CNCs in blue.

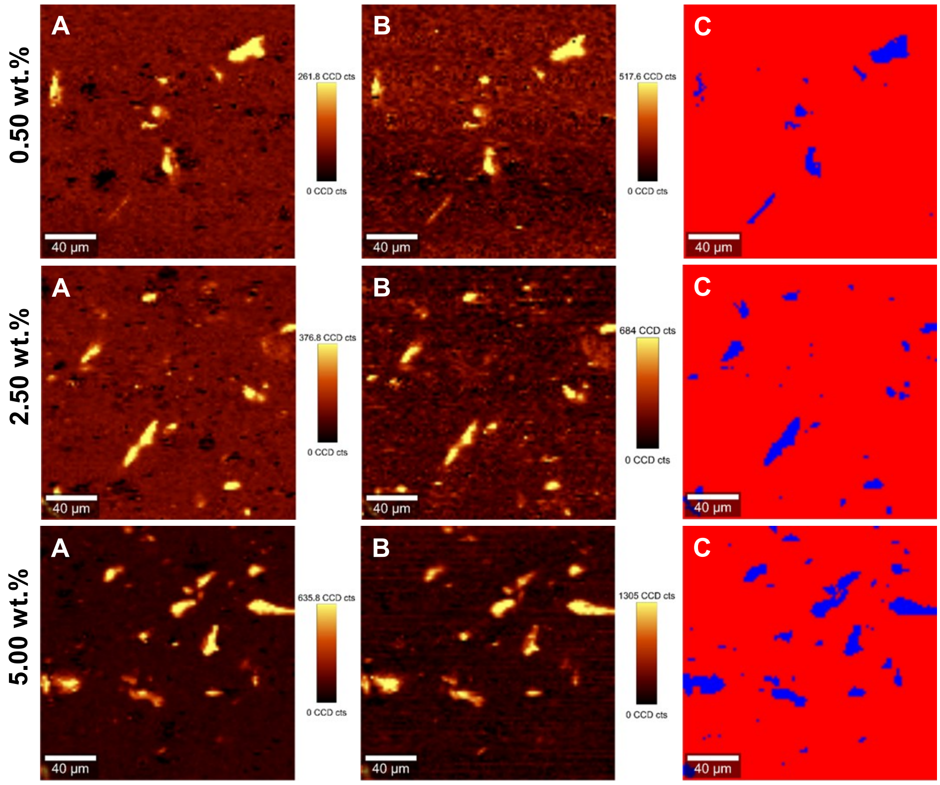

Figure S-3. Typical large area Raman images (LARI) of 0.50% (top), 2.50% (middle) and 5.00% (bottom) of CNCs/PEO/HDPE composites depicting the intensity of Raman bands located at ~380 cm^{-1} (A) and ~1095 cm^{-1} (B). Chemical images of studied area (C) showing HDPE in red and CNCs in blue.

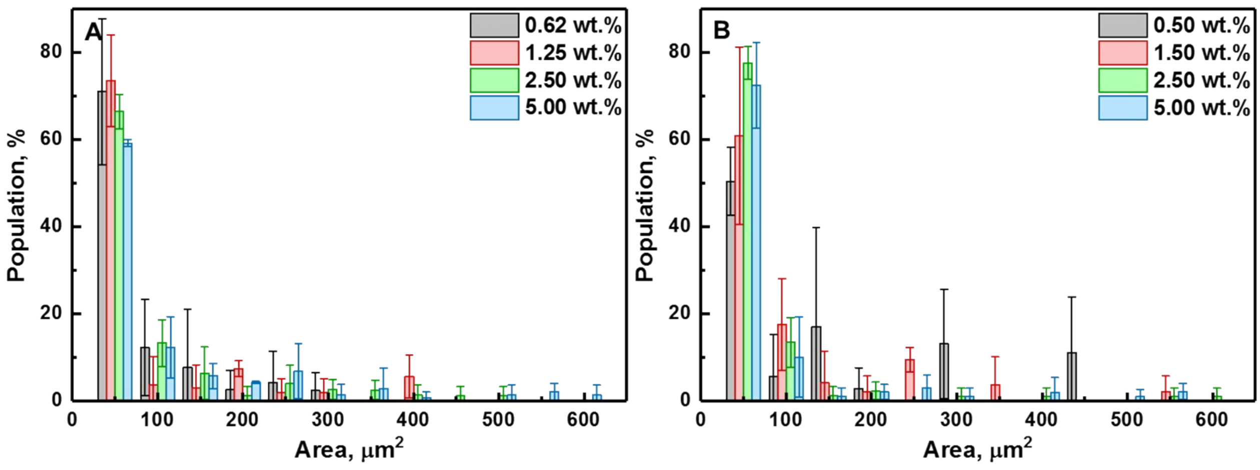

Figure S-4. Distribution of CNCs population vs. area of the CNCs aggregates for the CNCs/MAPE/HDPE (A) and CNCs/PEO/HDPE (B) composites. Results based on LARI Raman measurements.

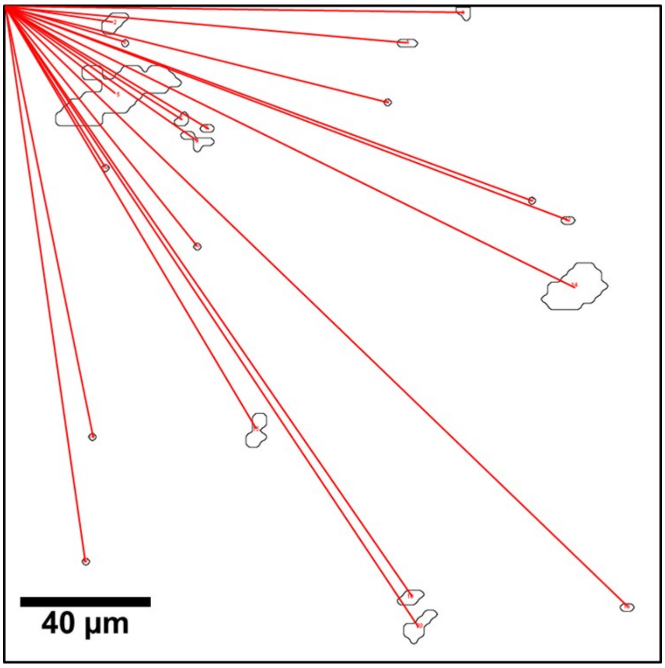

Figure S-5. Schematic representation of the ImageJ analysis of CNCs aggregates derived from the chemical image of 1.25% CNCs/MAPE/HDPE samples on Figure 3C. Distances (red lines) are calculated as $\sqrt{x^2 + y^2}$ from the x and y position of the center of mass of an aggregate (black outlines).

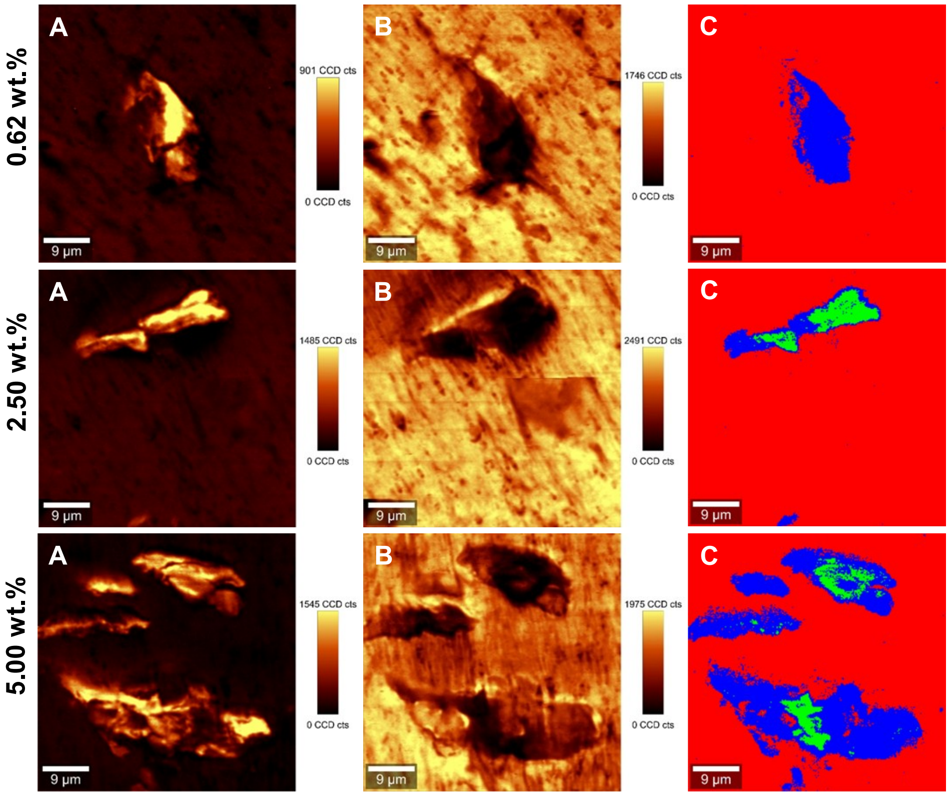

Figure S-6. Typical high-resolution Raman images (HRRI) of 0.62% (top), 2.50% (middle) and 5.00% (bottom) of CNCs/MAPE/HDPE composites depicting the intensity of Raman bands located at ~1100 cm^{-1} (A) and ~1301 cm^{-1} (B). Chemical images of studied area (C) showing the chemical composition of a mapped cross-section.

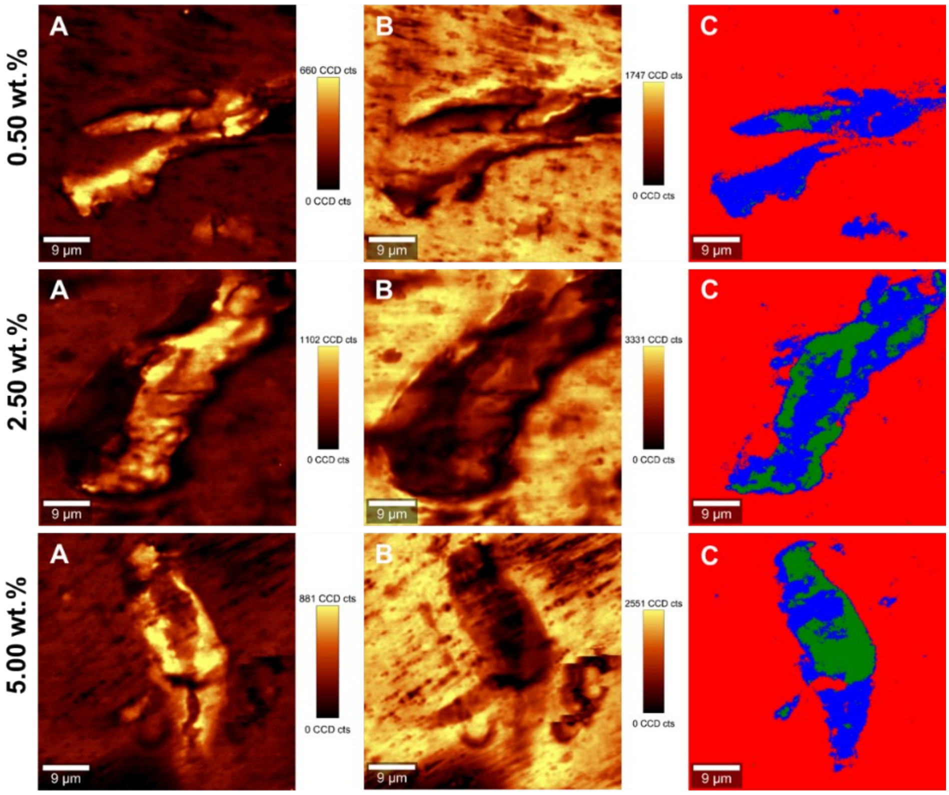

Figure S-7. Typical high-resolution Raman images (HRRI) of 0.50% (top), 2.50% (middle) and 5.00% (bottom) of CNCs/PEO/HDPE composites depicting the intensity of Raman bands located at ~1100 cm^{-1} (A) and ~1301 cm^{-1} (B). Chemical images of studied area (C) showing the chemical composition of a mapped cross-section.

Table S-1. Average distance of CNCs aggregates from an arbitrary point in the coordinate system, average size of CNCs aggregates and Raman based CNCs concentration for the series of CNCs/MAPE/HDPE and CNCs/PEO/HDPE.

Composite	Distance	Size	Concentration
	[μm]	[μm^2]	[wt.%]
0.62% CNCs/MAPE/HDPE	147 ± 9	53 ± 11	1.05 ± 0.33
1.25% CNCs/MAPE/HDPE	149 ± 10	84 ± 24	1.90 ± 0.63
2.50% CNCs/MAPE/HDPE	143 ± 7	70 ± 12	2.86 ± 0.97
5.00% CNCs/MAPE/HDPE	160 ± 5	111 ± 23	5.38 ± 1.55
0.50% CNCs/PEO/HDPE	135 ± 13	105 ± 27	1.26 ± 0.21
1.50% CNCs/PEO/HDPE	143 ± 7	69 ± 16	1.58 ± 0.74
2.50% CNCs/PEO/HDPE	150 ± 6	54 ± 13	2.53 ± 0.38
5.00% CNCs/PEO/HDPE	147 ± 6	149 ± 44	7.52 ± 3.10

Table S-2. Intensity of selected Raman bands and the intensity ratio used for description of the boundary of mixing degree levels.

Composite	Raman Band Intensity		Intensity Ratio
1.25% CNCs/MAPE/HDPE	1301 [cm^{-1}]	1381 [cm^{-1}]	1301/1381
Dark Green area	117	158	0.74
Green area	112	115	0.97
Lime area	144	117	1.23
Cyan area	162	50	3.23
Blue area	235	20	11.86
Navy area	355	13	26.88
1.50% CNCs/PEO/HDPE	1301 [cm^{-1}]	1285 [cm^{-1}]	1301/1285
Dark Green area	88	156	0.55
Green area	118	168	0.70
Lime area	227	201	1.13
Cyan area	312	191	1.63
Blue area	393	121	3.26
Navy area	578	68	9.14

Table S-3. Average area fraction of the component of chemical maps quantified for CNCs/MAPE/HDPE composites using Image J software.

Composite	Area fraction			Ratio of fraction	
	Red (R)* [μm^2]	Blue (B)* [μm^2]	Green (G)* [μm^2]	B/(R+B+G)	G/(R+B+G)
0.62% CNCs/MAPE/HDPE	2232±161	183±57	90±151	0.07±0.02	0.04±0.06
1.25% CNCs/MAPE/HDPE	2316±157	157±130	29±35	0.06±0.05	0.01±0.01
2.50% CNCs/MAPE/HDPE	2292±102	170±113	41±24	0.07±0.05	0.02±0.01
5.00% CNCs/MAPE/HDPE	2006±292	382±212	117±136	0.15±0.08	0.05±0.05

* Red – fraction area corresponding to HDPE; * Blue – fraction area corresponding to CNCs + HDPE; * Green - fraction area corresponding to CNCs

Table S-4. Average area fraction of the component of chemical maps quantified for CNCs/PEO/HDPE composites using Image J software.

Composite	Area fraction			Ratio of fraction	
	Red (R)* [μm^2]	Blue (B)* [μm^2]	Green (G)* [μm^2]	B/(R+B+G)	G/(R+B+G)
0.50% CNCs/PEO/HDPE	2135±43	248±146	120±123	0.10±0.06	0.05±0.05
1.50% CNCs/PEO/HDPE	2181±208	183±137	144±112	0.07±0.06	0.06±0.05
2.50% CNCs/PEO/HDPE	2139±237	215±173	150±87	0.09±0.07	0.06±0.03
5.00% CNCs/PEO/HDPE	1914±367	241±119	349±255	0.10±0.05	0.14±0.10

* Red – fraction area corresponding to HDPE; * Blue – fraction area corresponding to CNCs + PEO + HDPE; * Green - fraction area corresponding to CNCs + PEO
